# Concomitant High Apoptosis Inhibitor of Macrophage (AIM) and Low Prostate-Specific Antigen (PSA) Indicates Activated T Cell-Mediated Anticancer Immunity, Enhance Sensitivity to Pembrolizumab, and Elicit Good Prognosis in Prostate Cancer

**DOI:** 10.3390/biomedicines9091225

**Published:** 2021-09-15

**Authors:** Oluwaseun Adebayo Bamodu, Yuan-Hung Wang, Chi-Tai Yeh, Chen-Hsun Ho, Yi-Te Chiang, Wei-Tang Kao, Chia-Hung Liu, Chia-Chang Wu

**Affiliations:** 1 Department of Urology, Shuang Ho Hospital, Taipei Medical University, New Taipei City 235, Taiwan; 16625@s.tmu.edu.tw (O.A.B.); 11229@s.tmu.edu.tw (Y.-T.C.); 12319@s.tmu.edu.tw (W.-T.K.); mexxliu@tmu.edu.tw (C.-H.L.); 2 Department of Medical Research, Shuang Ho Hospital, Taipei Medical University, New Taipei City 235, Taiwan; d508091002@tmu.edu.tw (Y.-H.W.); ctyeh@s.tmu.edu.tw (C.-T.Y.); 3 Cancer Center, Department of Hematology and Oncology, Shuang Ho Hospital, Taipei Medical University, New Taipei City 235, Taiwan; 4 Graduate Institute of Clinical Medicine, College of Medicine, Taipei Medical University, Taipei 110, Taiwan; 5 TMU Research Center of Urology and Kidney (TMU-RCUK), Taipei Medical University, Taipei 110, Taiwan; 6 Department of Medical Laboratory Science and Biotechnology, Yuanpei University of Medical Technology, Hsinchu City 300, Taiwan; 7 Division of Urology, Department of Surgery, Shin Kong Wu Ho-Su Memorial Hospital, Taipei 111, Taiwan; m015695@ms.skh.org.tw; 8 School of Medicine, College of Medicine, Fu Jen Catholic University, New Taipei City 242, Taiwan; 9 Department of Urology, School of Medicine, College of Medicine, Taipei Medical University, Taipei 110, Taiwan

**Keywords:** prostate cancer, apoptosis inhibitor of macrophage, AIM, CD5L, prostate-specific antigen, PSA, KLK3, immune cells, T-cell, advanced disease, metastasis, recurrence, therapy-resistance, prognosis

## Abstract

Background: Despite its widespread use, the use of prostate-specific antigen (PSA) alone as a screening biomarker for prostate cancer (PCa) leads often to unwarranted prostate biopsy, over-diagnosis, and consequently, over-treatment, because of its limited specificity. There are reports that the apoptosis inhibitor of macrophage (AIM), secreted mainly by macrophages and epithelial cells, is upregulated during inflammation and facilitates immune recognition of cancerous cells by blocking human regulator of complement activation. Objective: These controversies around the PSA utility necessitate a reexamination of its use as a screening tool. More so, despite the suggested implication of AIM in anticancer immunosurveillance, there is a dearth of information on its role in therapy response, disease progression, and clinical outcomes of patients with PCa. These inform the present study to probe the nature and role of AIM/PSA signaling in anticancer immunity and prognosis in PCa. Methods: A combination of bioinformatics-aided statistical analyses, gene function annotation, and immune infiltrate analyses, coupled with tissue staining, and function assays, namely migration, invasion, and clonogenicity assays, we employed. Results: We demonstrated that AIM and PSA expression levels are inversely correlated in PCa clinical samples and cell lines, with AIM^low^PSA^high^ defining PCa, compared to AIM^high^PSA^low^ in normal samples. Concomitant aberrant PSA and significantly suppressed AIM expression levels positively correlated with high-grade disease and characterized by advanced stage prostate cancer, regardless of mutation status. We found that a high PSA/AIM ratio is associated with disease recurrence in patients with prostate cancer but is equivocal for overall survival. In addition, PSA-associated AIM suppression is implicated in the enhanced ‘metastability’ of PCa and a high AIM/PSA ratio is associated with strong castration-induced regression. CRISPR-mediated AIM knockout was associated with higher PSA expression while ectopic expression of AIM significantly attenuated the migration and invasive capability of PC3 and DU145 cells. Interestingly, compared to normal samples, we observed that AIM, biomarkers of T-cell activation and M1 phenotype markers are co-suppressed in PCa samples. Conclusion: Herein, we demonstrate that AIM/CD5L binds to PSA and that a high PSA/AIM ratio defines advanced stage PCa (regardless of mutation status), is implicated in enhanced metastability, and associated with disease recurrence, while a high AIM/PSA ratio is associated with strong castration-induced regression. More so, the ectopic expression of AIM significantly enhances the anticancer effect of Pembrolizumab and elicits an increased CD8+ T-cell count in AIM^hi^PSA^lo^PDL1+ PCa cases that are respondent to Pembrolizumab treatment.

## 1. Introduction

Prostate cancer (PCa) remains one of the most frequently diagnosed male malignancy and cause of cancer-specific mortality, globally, with an incidence of 7.1% and mortality rate of 3.8% in 2018 alone, and projected increase of ~1.8- and 2.1-fold in incidence and mortality, respectively, by the year 2040 [1]. The acquisition of a metastatic or recurrent phenotype by one in three PCa cases within 48 months from initial diagnosis, and evolution of a fifth of these into castration-resistant PCa (CRPC) by year five of follow up constitutes a clinical quagmire, especially in the context of about 14 months median survival for CRPC cases [2,3]. Unfortunately, coupled with this unabated disease incidence, enhanced risk of disease progression, and comparatively high mortality rate, our understanding of PCa biology and the molecular mechanisms of PCa pathogenesis and progression continue to evolve, remaining largely inconclusive.

Intracellular and genetic cancer-defining changes suggest a vital role for host T-cell-mediated anticancer immune response, wherein the immune cells recognize, target, and eradicate cancerous cells. Nonetheless, T-cell-mediated elimination of cancerous cells is only a part of the broad anticancer immunity cascade that ensures the intricate homeostasis of ‘self’ and ‘non-self’ recognition, thus, preventing autoimmunity, while honing precise targeting of malignant cells [4]. The identification of T-cell inhibitory signals, such as CTL4 and PDL1/PD1 in the tumor microenvironment (TME), has prompted the development of a whole new anticancer therapeutic strategy, namely, immunotherapy (more specifically, immune-checkpoint inhibition), which specifically represses the inhibition of immune effectors, while rejuvenating and broadening preexistent anticancer immune responses [4]. While immune-checkpoint inhibition (ICI) has triggered a paradigm shift in the treatment of malignancies, including PCa, and that emerging clinical data suggesting that cancer immunotherapy is quickly becoming a key component of contemporary clinical management of cancer, only a subset of patients exhibit durable response/remission, and ICI “has been less successful in treating prostate cancer than other solid tumors” [5,6], thus, necessitating a better understanding of the immune landscape in patients with PCa, identification of immune-related or immune-boosting biomarkers, and discovery of potential actionable therapeutic targets.

The prostate-specific antigen (PSA), also known as Kallikrein-related peptidase 3 (KLK3), is a single-chain glycoprotein and protease synthesized by the prostate epithelial cells and remains the most evaluated serum biomarker used for early prostate cancer screening, clinical staging, and therapy response monitoring. This is not without controversies. Most PSA—identified PCa are localized and usually low-grade disease, many of which never progress or become clinically relevant only after ~20 years [7]. This in itself raises the question of overdiagnosis of low-grade PCa, especially as the rapidly growing, difficult-to-treat, high-grade PCa are seldom found by PSA testing [7,8]. More so, being a highly sensitive biomarker, an abundance of false positives cannot be ruled out, and this is further compounded by the lack of consensual cut-off to delineate indolent from clinically relevant disease, thus yielding a pool of false positives and negatives [7,8]. For example, one in five CRPC cases that eventually respond to chemotherapy would have been labeled ‘non-responders’ based on initial persistent elevated PSA level, that did not decline on immune checkpoint blockade therapy or only declined after week 12 of chemotherapy [9]. This very modest or no association between changes in the post-treatment PSA level and treatment response or disease recurrence highlights a critical unmet need in PCa management. In light of the above, coupled with indismissible confounders such as benign prostate hypertrophy (BPH) and aging, the use of PSA as a standalone surrogate biomarker of cancerization, disease progression, or prognosis in patients with PCa, remains incongruous with available evidence, necessitating the discovery of supplementary PSA—related biomarkers. This need for improved biomarker reliability and accurate indicators of patient status, informs the present study.

The highly conserved scavenger receptors cysteine-rich (SRCR) superfamily consists of membrane-bound receptors that are essential for the recognition of both ‘self’ and ‘non-self’ targets, and play very vital roles in host defense, including pathogen sensing and cleaning [10]. The SRCR family integrates specific proteins “expressed by innate and adaptive immune cells for which no unifying function has yet been described” [11]. The Apoptosis inhibitor of macrophage (AIM/CD5L/API6/Spα) is a soluble/secreted member of the SRCR superfamily that is readily detected in the serum, varying with different clinical conditions [10,11]. In mice lacking AIM, the number of thymus cells was reduced by approximately 50% in comparison to their wild-type litter-mates, and interestingly, CD4^+^CD8^+^ thymus cells were found to be exceptionally more susceptible to dexamethasone or radiation-induced apoptosis, in vivo, conversely, apoptosis of the CD4^+^CD8^+^ thymus cells was markedly inhibited by ectopic AIM expression, in vitro [12]. This is suggestive of a role for AIM in inflammatory response regulation and immune cell viability. However, the probable role of AIM in the modulation of PSA activity, cancerization, disease progression, anticancer therapy response, and prognosis is largely unexplored.

The results presented herein demonstrate that AIM/CD5L binds to PSA, and that a high PSA/AIM ratio is characteristic of advanced-stage PCa (regardless of mutation status), implicated in enhanced metastability of PCa cells, associated with disease recurrence, while high AIM/PSA ratio is associated with strong castration-induced regression. More so, the ectopic expression of AIM was shown to significantly enhance the anticancer effect of Pembrolizumab and elicited an increased CD8^+^ T-cell count in AIM^hi^PSA^lo^PDL1+ PCa cases that were respondent to Pembrolizumab treatment.

## 2. Material and Methods

### 2.1. Prostate Cancer Tissue Samples

Prostate cancer tissue samples (*n* = 56) were obtained from the Shuang Ho Hospital, Taipei Medical University tissue bank after ethical approval from the Taipei Medical University Institutional Review Board (approval number: N202101071). The requirement for patients’ signed informed consent was waived because of the retrospective nature of the study.

### 2.2. Cell Culture and Chemicals

Androgen-independent metastatic prostate carcinoma PC-3 (ATCC^®^ CRL-1435™) and DU145 (ATCC^®^ HTB-81™) cell lines obtained from the ATCC (American Type Culture Collection, Manassas, VA, USA), were cultured in RPMI-1640 (Thermo Fisher Scientific Inc., Bartlesville, OK, USA) supplemented with 10% fetal bovine serum (FBS, #26140079, Thermo Fisher Scientific Inc., Bartlesville, OK, USA) and 100 U/mL of penicillin-streptomycin (Thermo Fisher Scientific Inc., Bartlesville, OK, USA). The cells were passaged when they attained ≥97% confluence and the culture medium was replenished every 48 h.

### 2.3. Drugs and Antibodies

Pembrolizumab (anti-PD-1; #A2005) was purchased from Selleck Chemicals (Houston, TX, USA). Stock solutions of 100 mM in 0.01% Dimethyl sulfoxide (DMSO; #D8418; Sigma-Aldrich Inc., St. Louis, MO, USA) were stored at −20 °C, until use. Monoclonal antibodies against CD5L/AIM (#sc-390486), KLK3/PSA (#sc-7316), and GAPDH (#sc-32233) was from Santa Cruz Biotechnology (Santa Cruz, CA, USA), while PDL1/CD274 (#14-5983-82; eBioscience™), was obtained from Thermo Fisher Scientific Inc. (Bartlesville, OK, USA).

### 2.4. Construction and Transfection of Plasmids Expressing AIM/CD5L

Ectopic expression of AIM/CD5L was achieved using the AIM (CD5L) (NM_005894) Human (Myc-DDK)-Tagged ORF Clone (#RC206528L2, OriGene Technologies, Inc., Rockville, MD, USA) in pCMV6-Entry vector transfected into PC-3 cells using Lipofectamine 2000 reagent (Invitrogen, Carlsbad, CA, USA) according to the manufacturer’s instructions. Cells transfected with empty vectors served as controls. Clones stably expressing AIM were selected by 100 mg/mL Ampicillin (#11593027, Thermo Fisher Scientific Inc., Bartlesville, OK, USA).

### 2.5. Cell Viability and Proliferation Colorimetric Assay

Cell viability was assessed using the Sulforhodamine B (SRB) assay. 3 × 10^3^ wild-type (WT) or CD5L-overexpressing (CD5L_OE) PC3 cells were seeded per well in 96-well microtiter plates containing supplemented growth media and incubated at 37 °C in humidified 5% CO_2_. After 48 h treatment with or without 5 μM Pembrolizumab, cell viability was measured following the manufacturer’s instructions. Briefly, the WT or CD5L_OE PC3 cells were fixed with 10% trichloroacetic acid (TCA), carefully washed with ddH_2_O, and then stained with 0.4:1 (*w*/*v*) SRB–acetic acid solution. Unbound SRB dye was washed off the cells with 1% acetic acid three times, plates containing stained cells were air-dried, and bound SRB dye solubilized using 10 mM Tris base. For cell proliferation, Invitrogen alamarBlue™ high sensitivity cell viability reagent (#A50100, Thermo Fisher Scientific Inc., Bartlesville, OK, USA) was used strictly following the manufacturer’s instruction. Briefly, after incubating WT or CD5L_OE PC3 cells with or without 5 μM Pembrolizumab in triplicates with three biological replicas for each assay at each time point (day 1–5), the cells were incubated with alamarBlue™ for 2 h at 37 °C, and the dye-stained viable/proliferating cells were quantified at an absorbance wavelength of 570 nm in the Molecular Devices Spectramax M3 multimode microplate reader (Molecular Devices LLC., San Jose, CA, USA).

### 2.6. Immunohistochemical (IHC) Staining Assays

Immunohistochemistry (IHC) analysis was performed on formalin-fixed paraffin-embedded (FFPE) sections from our PCa cohort consisting of clinical samples of different tumor grades (normal:Gleason score (GS) ≤ 5; low: GS = 6; medium: GS = 7; high: GS ≥ 8). The study was approved by the Taipei Medical University Institutional Review Board (approval number: N202101071) and performed following the recommendations from the Declaration of Helsinki for biomedical research involving human subjects. Antibodies against AIM/CD5L, PSA/KLK3, and PDL1/CD274 were used at 1:250 dilution following standard IHC protocol. For protein expression scoring, automated scoring was adopted using the National Institutes of Health ImageJ software version 1.49 (https://imagej.nih.gov/ij/, accessed on 27 July 2021), with confirmation by two independent pathologists. For cell visualization and imaging, the Nikon E800 fluorescent microscope (Nikon Instruments Inc., Melville, NY, USA) was used.

### 2.7. Scratch Wound-Healing Migration Assay

To assess cell migration, the scratch wound-healing assay was performed. Briefly, WT or CD5L_OE PC3 and DU145 cells were seeded and allowed to grow in 6-well plates (Corning, Corning, NY, USA) containing complete growth media with 10% FBS. Once the cells attained >98% confluence, the media in the wells was changed to low serum (1% FBS) growth media. The median axes of the adherent mono-layered PCa cells were denuded with sterile yellow pipette tips. Cell migration based on wound closure was monitored over time, and images were captured at 0 and 18 h after denudation, under a light microscope using a 10X objective lens. Thereafter, the images were analyzed using National Institutes of Health ImageJ software version 1.49 (https://imagej.nih.gov/ij/, accessed on 27 July 2021).

### 2.8. Invasion Assay

For invasion assay, we used the Corning^®^ BioCoat^TM^ Matrigel^®^ Invasion Chambers with 8.0 μm PET Membrane in two 24-well plate systems (#354480, Corning, Corning, NY, USA). 1 × 10^5^ WT or CD5L_OE PC3 cells treated with or without 5 μM Pembrolizumab were seeded per well in the plates and incubated overnight at 4 °C. The upper chambers contained 2% FBS-supplemented (low serum) media whereas the lower chamber contained 600 μL 20% FCS-supplemented (high serum) media. After incubating for 48 h, the non-invaded cells in the upper chamber were wiped off carefully with sterile cotton buds, while the cells that invaded/penetrated through the membrane to the lower chamber were fixed with ethanol, stained with crystal violet dye, and counted under a light microscope from five randomly selected fields of vision.

### 2.9. Public Cancer Dataset Access and Analysis

The public online cancer data repositories used in this study include a curated dataset across 32 cancer types (*n* = 20,386) from the Institute of Cancer Research (ICR) online platform [13]. The broad_2020_09_c7_immunologic dataset on the R2: Genomics Analysis and Visualization Platform (https://hgserver1.amc.nl/cgi-bin/r2/main.cgi, accessed on 22 February 2021), MET500 metastatic PRAD cohort data on whole-exome and transcriptome sequencing of ~500 metastatic cancer samples (MET500) [14,15]. We also probed GSE40272 (HG-U133_Plus_2) Affymetrix Human Genome U133 Plus 2.0 Array dataset (*n* = 98), GSE46691 (HuEx-1_0-st) Affymetrix Human Exon 1.0 ST Array dataset (*n* = 545), GSE7930 (HG-U133) Affymetrix Human Genome U133 Array dataset (*n* = 6), GSE21887 (HG-U133_Plus_2) Affymetrix Human Genome U133 Plus 2.0 Array dataset (*n* = 12), and GSE32269 (HG-U133) Affymetrix Human Genome U133 Array dataset (*n* = 55) using the National Center for Biotechnology Information Gene Expression Omnibus (NCBI GEO) data Browser (https://www.ncbi.nlm.nih.gov/geo/query/acc.cgi?, accessed on 15 February 2021) and the Oncomine (https://www.oncomine.org/resource/main.html#v:18, accessed on 02 March 2021) online platforms. Gene ontology analysis was performed using the NIAID/NIH DAVID Bioinformatics Resources software version 6.8 (https://david.ncifcrf.gov/, accessed on 19 February 2021).

### 2.10. Statistical Analysis

All data are representative of the mean ± standard deviation (SD) of assays performed three times in triplicates. For comparison between two groups, we used the two-sided Student’s *t*-test, and one-way analysis of variance (ANOVA) with Tukey’s post hoc test aided comparison between ≥3 groups. Kaplan-Meier survival analyses were used to compare survival rates between groups. All statistical analyses were performed using GraphPad Prism version 8.0.0 for Windows (GraphPad Software, La Jolla, CA, USA). *p*-value < 0.05 was considered statistically significant.

## 3. Results

### 3.1. AIM and PSA Expression Levels Are Inversely Correlated in Patients with Prostate Cancer

**Figure 1:**
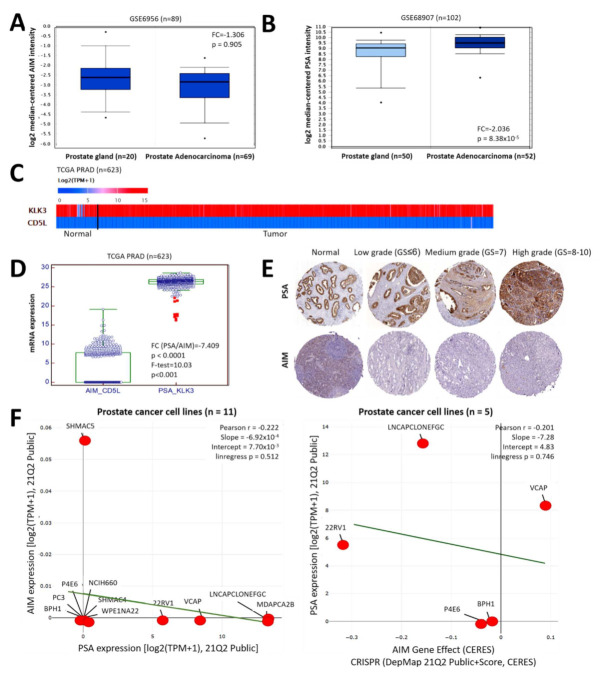
AIM and PSA expression levels are inversely correlated in patients with prostate cancer. Box and whisker plots of the differential expression of (**A**) *AIM/CD5L* and (**B**) *PSA/KLK3* transcript expression in the prostate gland or prostate adenocarcinoma from the GSE6956 and GSE68907 cohort, respectively. (**C**) Comparative heatmap of the expression of *KLK3* and *CD5L* in TCGA PRAD normal and tumor samples. (**D**) Box and whisker plots comparing the mRNA expression levels of *AIM/CD5L* and *PSA/KLK3* in the TCGA PRAD cohort. (**E**) Representative IHC images showing the differential expression of AIM and AIM in normal prostate, low-grade, medium grade, and high-grade PCa samples from the TMU-SHH cohort. (**F**) Line and dots plot of the correlation between AIM and PSA transcript expression in prostate cancer cell lines from the DepMap Public 21Q2 dataset (**left**). Line and dot plot showing the effect of CRISPR-mediated *aim* gene knockout, on PSA transcript expression in prostate cancer cell lines from the DepMap Public 21Q2 dataset (**right**). TCGA, the cancer genome atlas; PRAD, prostate adenocarcinoma; IHC, immunohistochemistry; TPM, transcripts per million.

Initial expression profile probe of the GSE6956 PCa array data (*n* = 89) showed that the expression of *AIM* transcript in ‘normal’ prostate gland samples was higher than in the PCa samples (I.31-fold, *p* = 0.91) (Figure 1A). On the other hand, analysis of the GSE68907 PCa dataset (*n* = 102) showed that compared to the normal samples, *PSA* transcript expression was significantly upregulated in the PCa samples (2.04-fold, *p* = 8.38 × 10^−5^) (Figure 1B). Re-analysis of the TCGA PRAD cohort data (*n* = 623) showed that regardless of sample type, the expression of *AIM/CD5L* and *PSA/KLK3* is inversely correlated (Figure 1C), such that relative to *AIM*, *PSA* transcript expression was significantly overexpressed (7.41-fold, *p* < 0.0001) (Figure 1D). Immunohistochemistry analysis of our in-house PCa samples (*n* = 56), showed that while the expression of PSA protein increased with higher tumor grade, AIM expression was strong in the normal prostate samples but a very mild or null expression was observed in PCa samples, regardless of tumor grade (Figure 1E). In corroboration, using the cancer dependency map (https://depmap.org/portal/interactive/, accessed on 31 January 2021) SHMAC5, P4E6, NCIH660, PC3, BPH1, SHMAC4, WPE1NA22, 22RV1, VCAP, LNCaP clone FGC, and MDAPCA2B PCa cell lines data, we found an inversely corrected expression pattern between *AIM* and *PSA* transcripts (Pearson r = −0.22, Slope = −0.00069, linregress *p* = 0.51) (Figure 1F, left). Furthermore, in the context of their inversely correlated expression, we probed for likely genetic dependencies between AIM and PSA. Using the cancer dependency map platform (https://depmap.org/portal/interactive/, accessed on 30 January 2021), we observed that CRISPR-induced suppression of *aim/cd5l* gene effect, elicited upregulated *PSA* transcript expression in LNCaP clone FGC, 22RV1, VCAP, P4E6, and BPH1 PCa cell lines (Pearson r = −0.20, Slope = −7.28, linregress *p* = 0.75) (Figure 1F, right). These data indicate, at least in part, that AIM and PSA expression levels are inversely correlated in patients with prostate cancer.

### 3.2. Aberrantly Expressed PSA and Significantly Suppressed AIM Expression Characterize Advanced Stage Prostate Cancer, Regardless of Mutation Status

**Figure 2:**
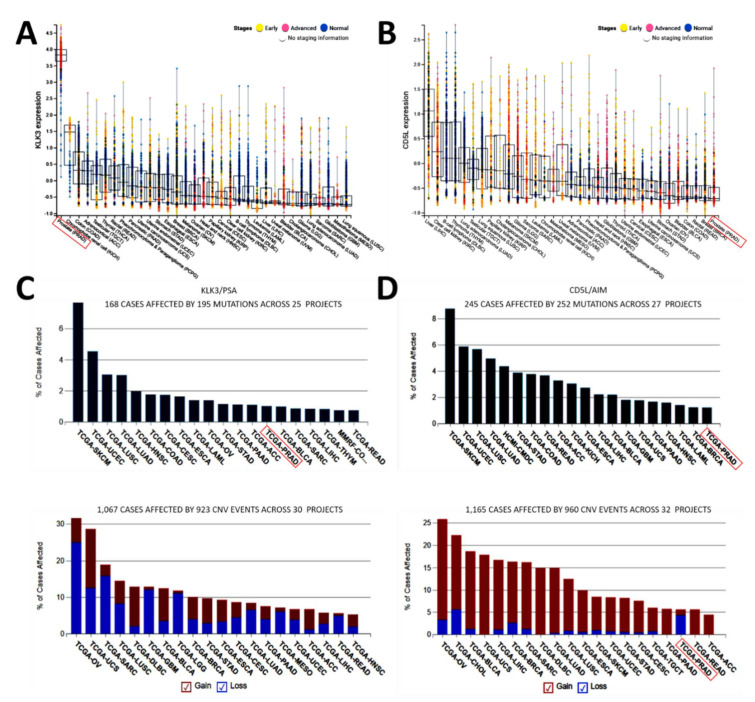
Aberrantly expressed PSA and significantly suppressed AIM expression characterize advanced stage prostate cancer, regardless of mutation status. Box and whisker and dot plots of the expression profiles of (**A**) PSA/KLK3 and (**B**) AIM/CD5L in normal tissue, early stage, or advanced stage disease across 32 cancer types from the ICR-curated dataset. Graphical representation of cases affected by (**C**) *psa/klk3* and (**D**) *aim/cd5l* mutation (**upper**) or CNV (**lower**) in ICR-curated pan-cancer TCGA cohort data. ICR, Institute of Cancer Research; CNV, copy number variation; TCGA, the cancer genome atlas; PRAD, prostate adenocarcinoma; red box, PCa/PRAD cohort.

To gain some insight into the functional relevance of the observed association between AIM and PSA expression, we evaluated the expression profile of PSA/KLK3 across 32 cancer types (*n* = 20,386) at different clinical stages using the Institute of Cancer Research (ICR)-curated dataset [13]. Our results indicate that PSA expression was highest in the PCa (prostate adenocarcinoma, PRAD) sample; More interestingly, per stage, PSA expression was highest in patients with advanced stage PCa, compared to the early stage or normal cases (Figure 2A). Conversely, we found that in this pan-cancer cohort, the least expression of AIM was found in the PCa/PRAD samples, and more so in the advanced stage cases (Figure 2B). Because of the implication of gene mutation on its function [14], we queried the same ICR cancer dataset and found that the *psa/klk3* mutation in PCa/PRAD accounted for ~1.4% of the 168 cases affected by 195 mutations across 25 projects (Figure 2C). On the other hand, we found that while ~1.0% of 245 cases affected by 252 mutations across 27 projects was attributable to *aim/cd5l* gene mutation in PRAD, these were associated mainly with the loss of gene function copy number variation (CNV) (Figure 2D), thus explaining the very mild or null expression of AIM in Figure 1E. These data indicate that aberrantly expressed PSA and significantly suppressed AIM expression characterize advanced stage prostate cancer, regardless of mutation status.

### 3.3. The Differential Expression of PSA and AIM Is Associated with Disease Recurrence in Patients with Prostate Cancer but Is Equivocal for Overall Survival

**Figure 3:**
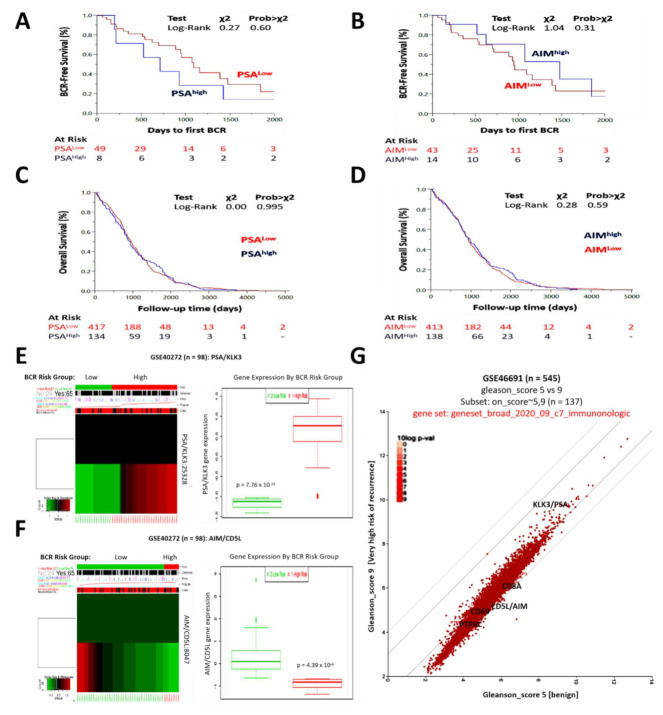
The differential expression of PSA and AIM is associated with disease recurrence in patients with prostate cancer but is equivocal for overall survival. Kaplan–Meier curves showing the effect of high or low (**A**) PSA and (**B**) AIM expression on BCR-free survival in the TCGA PRAD cohort. Kaplan–Meier curves showing the effect of high or low (**C**) PSA and (**D**) AIM expression on overall survival in the TCGA PRAD cohort. BCR risk group-stratified heatmap (**left**) and box-and-whisker plots (**right**) of (**E**) *PSA/KLK3* or (**F**) *AIM/CD5L* expression levels in the GSE40272 cohort. (**G**) XY plot showing the differential expression of AIM and PSA in the GSE within the Broad_2020_09_c7_immunologic gene set. BCR, biochemical recurrence; TCGA, the cancer genome atlas; PRAD, prostate adenocarcinoma.

We further explored the clinical relevance of PSA and AIM expression using the TCGA PRAD cohort (*n* = 623), and found that compared to patients with low PSA expression, patients with high PSA expression exhibited worse biochemical recurrence-free (BRF) survival (*X*^2^ = 0.27, Prob > *X*^2^ = 0.60) (Figure 3A). Conversely, patients with high AIM expression enjoyed a BRF survival advantage compared to their low AIM peers (*X*^2^ = 1.04, Prob > *X*^2^ = 0.31) (Figure 3B). However, we observed and find it intriguing that the differential expression of PSA or AIM was equivocal for overall survival in the TCGA PRAD cohort (Figure 3C,D). Furthermore, reanalysis of the GSE40272 PCa cohort data (*n* = 98) showed that patients with high *psa/klk3* expression had an elevated prognostic index, and were at significantly higher risk of biochemical recurrence (BCR), compared to the low expressors (2.01-fold, *p* = 7.76 × 10^−24^) (Figure 3E). However, patients with markedly suppressed *aim/cd5l* expression were at higher risk of BCR compared to those with significantly upregulated *aim/cd5l* expression, which were wholly in the low-risk group (*p* = 7.76 × 10^−24^) (Figure 3F). In the validation analysis of the Jenkins’ GSE46691 dataset (*n* = 545) originally on genomic classifiers for identification of aggressive prostate cancer, we found that compared with their non-tumor peers (gleason score 5), patients at very high risk of early metastatic recurrence (gleason score 9) were genotypically AIM^low^PSA^high^CD8A^low^CD69^low^PTPRC^low^ using the broad_2020_09_c7_immunologic dataset on the R2: Genomics Analysis and Visualization Platform (https://hgserver1.amc.nl/cgi-bin/r2/main.cgi, accessed on 26 February 2021) (Figure 3G). These data indicate that the differential expression of PSA and AIM is associated with disease recurrence in patients with PCa, and has an immunologic undertone.

### 3.4. PSA-Associated Suppression of AIM Is Implicated in the Enhanced Metastability of Prostate Cancer and a High AIM/PSA Ratio Is Associated with Strong Castration-Induced Regression

**Figure 4:**
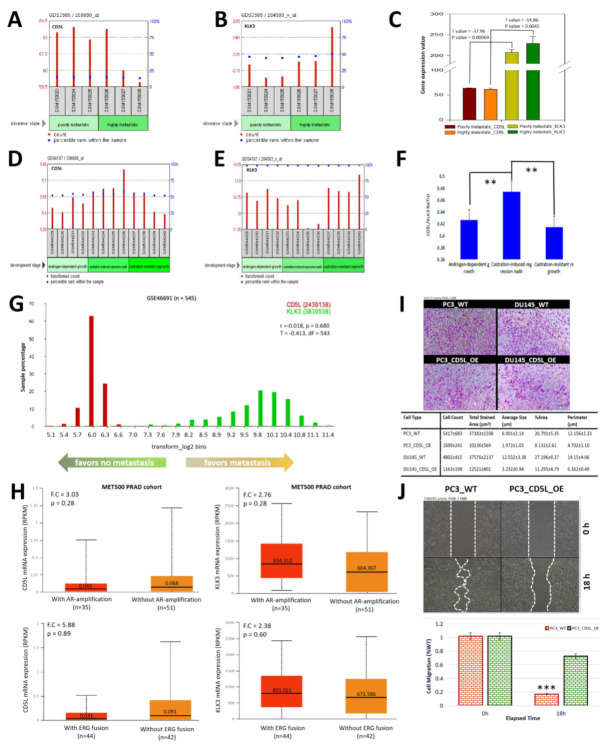
PSA-associated suppression of AIM is implicated in the enhanced metastability of prostate cancer and a high AIM/PSA ratio is associated with strong castration-induced regression. Histograms showing the differential expression of (**A**) *cd5l/aim* and (**B**) *klk3/psa* in poorly metastatic or highly metastatic sample from the GDS2865 dataset. (**C**) Histogram comparing the *cd5l* and *klk3* expression values in the poorly or highly metastatic samples from the GDS2865 dataset. Histograms showing the differential expression of (**D**) *cd5l/aim* and (**E**) *klk3/psa* in androgen-dependent growth, castration-induced regression nadir, or castration-resistant regrowth samples from the GDS4107 dataset. (**F**) Histograms of the *cd5l/klk3* ratio in androgen-dependent growth, castration-induced regression nadir, or castration-resistant regrowth samples from the GDS4107 dataset. (**G**) Sample percentage bin plot of *cd5l* and *klk3* expression in the GSE46691 cohort stratified by propensity for metastasis. (**H**) Box and whisker plots of the differential expression levels of *CD5L* (**upper left**) and *KLK3* (**upper right**) in patients with or without AR-amplification in the MET500 PRAD cohort. Box and whisker plots of the differential expression levels of *CD5L* (**lower left**) and *KLK3* (**lower right**) in patients with or without ERG fusion in the MET500 PRAD cohort. (**I**) Representative photomicrographs (**upper**) and quantitative chart (**lower**) showing the effect of CD5L_OE on invasion of PC3 or DU145 cells. (**J**) Representative photomicrographs (**upper**) and histograms (**lower**) showing the effect of CD5L_OE on migration of PC3 cells at indicated time-points. CD5L_OE, overexpression of *cd5l*; AR, androgen receptor; F.C., fold change; ** *p* < 0.01; *** *p* < 0.001.

To understand the role of PSA and/or AIM in the metastatic dissemination of PCa cells and disease progression, we analyzed the GSE7930/GPL96/GDS2865 Affymetrix Human Genome U133A Array dataset that originally compared poorly and highly metastatic prostate subcutaneous tumors. Unlike *aim/cd5l* expression, which is upregulated in poorly metastatic PCa cells and suppressed in the highly metastatic PCa (Figure 4A), our results showed that *psa/klk3* expression is downregulated in the poorly metastatic in comparison to its upregulated expression in the highly metastatic samples (Figure 4B). In a parallel analysis, regardless of the degree of metastability, we found that the expression of *aim/cd5l* was lower and *psa/klk3* higher, with a 1.29-fold decrease in the AIM/PSA ratio in the highly metastatic relative to the poorly metastatic cells (*p* < 0.001) (Figure 4C). Furthermore, our re-analysis of the GSE21887/GPL570/GDS4107Affymetrix Human Genome U133 Plus 2.0 Array originally analyzing samples from androgen-dependent growth (AD), castration-induced regression nadir (ND), and castration-resistant regrowth (CR) stages to provide insight into the molecular basis of castration-resistant prostate cancer (CRPC) development, showed that the expression of *aim/cd5l* was upregulated in the ND samples compared to the AD and CR samples (Figure 4D), while *psa/klk3* expression was lowest in the ND samples, relatively upregulated in the AD samples, and highest in the CR group (Figure 4E). Compared with the AD and CR samples, we found the AIM/PSA ratio increased by 1.11-fold (*p* < 0.01) and 1.13-fold (*p* < 0.01), respectively (Figure 4F). We also probed the GSE46691 data (*n* = 545) initially on the discovery and validation of prostate cancer genomic classifiers that predict early metastasis following radical prostatectomy. Our results showed that upregulated expression of *AIM/CD5L* transcripts favored “no metastasis”, while upregulated *PSA/KLK3* expression favored “metastasis” (T = −0.413, r = −0.018, *p* = 0.68) (Figure 4G). Next, cognizant of the culpability of androgen receptor (AR) signaling or TMPRSS2:ERG gene fusion in PCa metastasis and disease progression, we re-analyzed Robinson et al.’s data on whole-exome and transcriptome sequencing of ~500 metastatic cancer samples (MET500) [15], and showed that *AIM/CD5L* mRNA expression was 1.51-fold (*p* = 0.28) upregulated in the PCa samples without AR-amplification, compared to the AR-amplified samples from the MET500 metastatic PRAD cohort (Figure 4H, upper left). Conversely, we observed a 1.38-fold (*p* = 0.28) increased *PSA/KLK3* expression in the samples with AR-amplification, compared to those without AR-amplification (Figure 4H, upper right). We also demonstrated that *AIM/CD5L* expression was enhanced in the “without ERG fusion” group, compared to those with ERG fusion (2.94-fold, *p* = 0.89), while *PSA/KLK3* transcript expression was upregulated in the “with ERG fusion” group, compared to their peers without (1.19-fold, *p* = 0.60) (Figure 4H, lower). In corroboratory in vitro analyses using the ectopic expression of AIM in PC3 or DU145 cells (CD5L_OE), we demonstrated a 3.19-fold (*p* < 0.005) and 4.13-fold (*p* < 0.01) decrease in the number of invaded PC3_CD5L_OE and DU145_CD5L_OE cells, in comparison to their wild type (WT) counterparts (Figure 4I). More so, we found that CD5L_OE significantly attenuated the migration of in the PC3_CD5L_OE cells, relative to the PC3_WT cells (3.68-fold, *p* < 0.001) (Figure 4J). These data indicate that PSA-associated suppression of AIM is implicated in the enhanced metastability of prostate cancer and a high AIM/PSA ratio is associated with strong castration-induced regression.

### 3.5. The Functional Association between PSA and AIM Modulates Metastasis and Mirrors the Immunogenicity in Patients with Prostate Cancer

**Figure 5:**
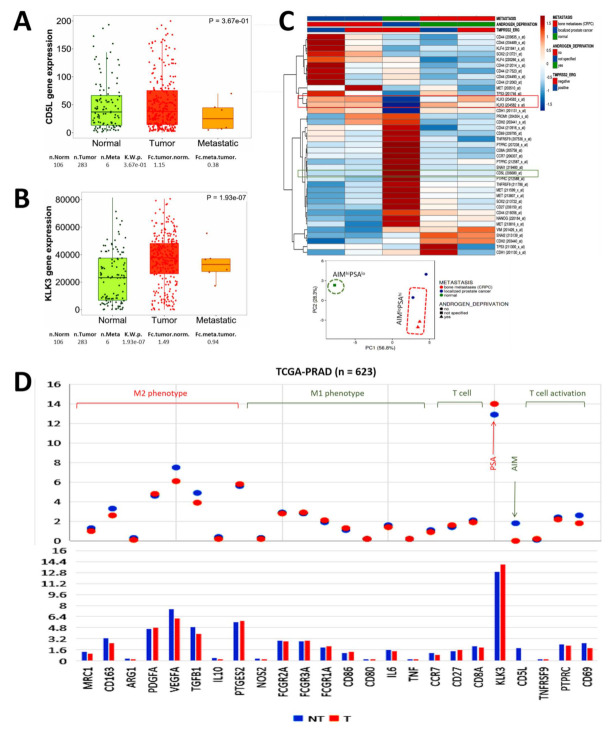
The functional association between PSA and AIM modulates metastasis and mirrors the immunogenicity in patients with prostate cancer. Box and whisker plots showing the differential expression of (**A**) *cd5l/aim* and (**B**) *psa/klk3* genes normal, primary tumor, or metastatic samples from the TNMplot gene chip data. (**C**) Expression heatmap showing the correlation between *CD5L*, *KLK3*, with biomarkers of cancer stemness, epithelial-to-mesenchymal transition, metastasis, or T-cell activation in normal, localized PCa, and bone metastatic CRPC in the GSE32269. (**D**) Graphical representation of the differential expression of *CD5L*, *KLK3*, biomarkers of M1 phenotype, M2 phenotype, and T-cell activation in non-tumor or tumor samples from the TCGA PRAD cohort.

Corroboratory results from the TNMplot gene chip data (*n* = 56,938) [16] showed that compared to the normal samples, the median expression of *aim/cd5l* was 2.30- and 3.06-fold lower in the tumor and metastatic samples, respectively (*p* = 0.37) (Figure 5A). On the other hand, *psa/klk3* expression was 2.98- and 1.23-fold higher in the tumor and metastatic samples, respectively (*p* = 1.93 × 10^−7^) (Figure 5B). Re-analysis of GSE32269 expression data for primary localized prostate cancer versus castration-resistant bone metastatic PCa (*n* = 55) confirmed that compared to the downregulated expression of *AIM/CD5L* transcripts in localized PCa and castration-resistant bone metastatic PCa, regardless of TMPRSS2-ERG fusion status, upregulation of *PSA/KLK3* expression was observed in both localized and castration-resistant bone metastatic PCa; Interestingly, in the normal samples, while *PSA/KLK3* expression was suppressed, the expression of *AIM/CD5L* was significantly enhanced (Figure 5C). Cognizant of the crosstalk between cancerous and immune cells, as well as the complicity of this interaction in metastasis, we re-probed the TCGA PRAD cohort data (*n* = 623) and found that compared to normal samples, there was concomitant downregulation of *AIM/CD5L*, biomarkers of T-cell activation namely *CD8A*, *CCR7* (C-C motif chemokine receptor 7), *PTPRC* (protein tyrosine phosphatase receptor type C), *CD69*, and M1 phenotype markers *IL6* (interleukin 6), *FCGR2A* (Fc fragment of IgG receptor IIa), *FCGR3A*, *NOS2* (nitric oxide synthase 2) in the PCa samples, while M2 macrophage-associated platelet-derived growth factor subunit A (*PDGFA*) and prostaglandin E synthase 2 (*PTGES2*) were upregulated (Figure 5D). These data, at least in part, indicate that the functional association between PSA and AIM modulates metastasis and mirrors the immunogenicity in patients with PCa.

### 3.6. The Inversely Correlated Expression of PSA and AIM Differentially Modulate Treg, T cell, and Macrophage Activities in Patients with Prostate Cancer

**Figure 6:**
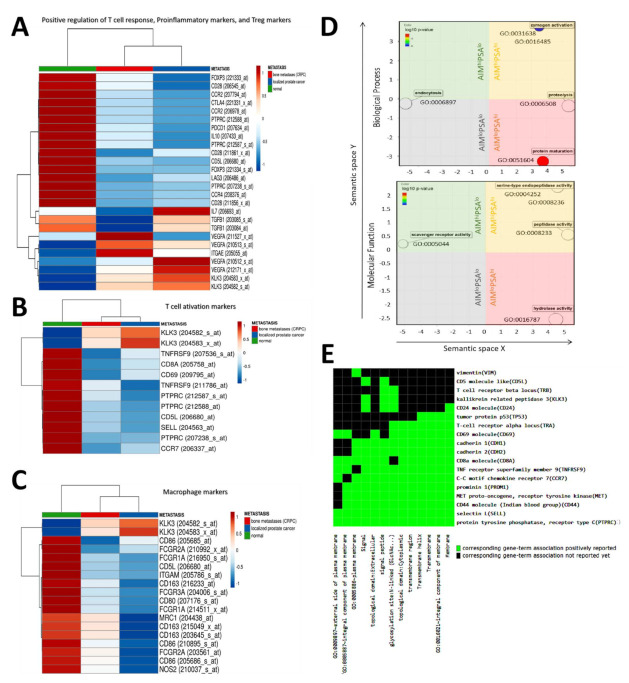
Heatmap showing the correlation between *CD5L*, *KLK3*, with (**A**) biomarkers of positive regulation of T-cell responses, pro-inflammatory markers, Treg markers, (**B**) T-cell activation markers, and (**C**) macrophage markers, in normal, localized PCa, and bone metastatic CRPC in the GSE32269. Columns with similar annotations are collapsed by taking mean inside each group. Rows are centered; unit variance scaling is applied to rows. Both rows and columns are clustered using correlation distance and average linkage. Varied rows, three columns. (**D**) Graphical visualization of the five clusters formed by *cd5l* and *klk3* gene-enrichment-based molecular or biological process functional annotation clustering using a medium classification stringency. (**E**) Gene ontology visualization of reported gene-term association. Treg, regulatory T cell; CRPC, castration-resistant prostate cancer.

To understand the nature of immunogenicity elicited by or associated with AIM/PSA signaling, we re-analyzed the GSE32269 expression data for primary localized prostate cancer versus castration-resistant bone metastatic PCa (*n* = 55). Our results showed that relative to downregulated *AIM/CD5L* expression, *PSA/KLK3* is co-upregulated with regulatory T-cell (Treg) markers *IL7*, and *VEGFA* in localized PCa, or with *VEGFA*, and *ITGAE* in castration-resistant bone metastatic PCa (Figure 6A). However, while *PSA/KLK3* was suppressed, *AIM/CD5L* was concurrently upregulated with biomarkers of positive T-cell response *CD28*, *CCR2*, *PTPRC*, and Treg markers *IL10*, *PDCD1*, *FOXP3*, *LAG3*, *CCR4*, in normal samples (Figure 6A). Interestingly, different isoforms of *TGFB1* were upregulated in normal, localized PCa and castration-resistant bone metastatic PCa. In a parallel analysis, we found that, unlike *PSA/KLK3*, which was upregulated, *AIM/CD5L* was co-suppressed with markers of T-cell activation *CD8A*, *TNFRSF9*, *CD69*, *PTPRC*, *SELL*, and *CCR7* mRNA in primary localized and castration-resistant bone metastatic PCa, but markedly enhanced in normal samples (Figure 6B). Furthermore, we observed that in bone metastatic CRPC, *AIM/CD5L,* and M1 macrophage markers *CD86, FCGR2A, FCGR1A, ITGAM, FCGR3A, CD80*, were co-downregulated, while *PSA/KLK3,* and M2 macrophage markers *MRC1/CD206, CD163* were concurrently upregulated (Figure 6C). In addition, *PSA/KLK3* was upregulated, but *AIM/CD5L* downregulated in localized PCa, while the reverse was the case in normal samples (Figure 6C). For better characterization of the roles of AIM/PSA signaling in PCa, we performed a gene ontology analysis using the NIAID/NIH DAVID Bioinformatics Resources software version 6.8 (https://david.ncifcrf.gov/, accessed on 20 February 2021). Our functional annotation clustering showed the AIM and PSA concertedly play essential roles in certain biological processes, namely, protein maturation (GO:0051604), proteolysis (GO:0006508), zymogen activation (GO:0031638), and protein processing (GO:0016485), through their hydrolase (GO:0016787), peptidase (GO:0008233), serine-type endopeptidase (GO:0004252), serine-type peptidase (GO:0008236), and scavenger receptor (GO:0005044) activities (Figure 6D). Moreover, gene enrichment-function association studies showed that AIM/CD5L, PSA/KLK3, T-cell receptor β locus, CD8A, TNFRSF9, CCR7, SELL, PTPRC, CD24, CD44, PROM1, CDH1, CDH2, and MET are enriched for ‘signal’ and ‘signal peptides’ which are essential for protein secretion and sorting [17], while all the above but AIM/CD5L and CD8A, plus TRA, and CD69, are associated with N-linked glycosylation, a stringent mechanism of intracellular secondary protein processing which is essential for protein structure, function, and stability determination, as well as a cellular response to exogenous factors [18]. These data indicate, at least in part, that the inversely correlated expression of PSA and AIM differentially modulate Treg, T-cell, and macrophage activities in patients with prostate cancer.

### 3.7. AIM Binds Directly to PSA, and the AIM-PSA Interactome Reveals Complicity in Immune Landscape, Macrophage, EMT and CSC Regulation

**Figure 7:**
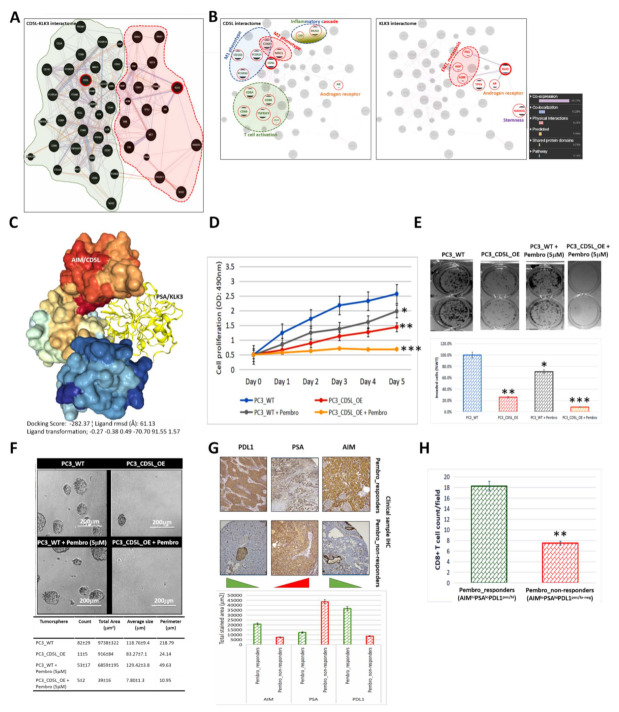
AIM binds directly to PSA, and the ectopic (re)expression of AIM enhances the anticancer therapeutic effect of Pembrolizumab against PCa cells. 2D visualization of the auto-generated (**A**) CD5L-KLK3 interactome showing complicity in the immune landscape, macrophages, EMT and CSC regulation, (**B**) CD5L interactome, or KLK3 interactome. (**C**) Three-dimensional visualization of the direct complex formation by AIM/CD5L (surface model) and PSA/KLK3 (ribbon model). (**D**) Line graph showing the effect of CD5L_OE with or without 5 μM Pembrolizumab on PC3 cell proliferation. (**E**) Representative photomicrographs (**upper**) and histograms (**lower**) showing the effect of CD5L_OE with or without 5 μM Pembrolizumab on PC3 cell invasion. (**F**) Representative photomicrographs (**upper**) and quantitative chart (**lower**) showing the effect of CD5L_OE with or without 5 μM Pembrolizumab on the tumorsphere formation capability of PC3 cells. (**G**) Representative IHC photomicrographs (**upper**) and histograms (**lower**) of the immunoreactivity of AIM, PSA, and PDL1 in pembro_responders or pembro_non-responders from the TMU-SHH cohort. (**H**) Histograms showing the differential CD8^+^ T-cell count/field in pembro_responders or pembro_non-responders from the TMU-SHH cohort. WT, wild type; OE, overexpression; * *p* < 0.05; ** *p* < 0.01; *** *p* < 0.001.

To gain some mechanistic insight into the role of AIM/PSA signaling in PCa initiation, immune response, and progression, we performed an automated generation of AIM-PSA interaction networks based on functional enrichment. We found markers of epithelial-to-mesenchymal transition (EMT) and metastasis, cadherin 1 (CDH1), snail (SNAI1), slug (SNAI2), vimentin (VIM), hepatocyte growth factor (HGF), MET, catenin delta 1 (CTNND1), cbl proto-oncogene (CBL), fibronectin 1 (FN1), anti-inflammatory signal alpha-2-macroglobulin (A2M), stemness markers kruppel-like factor 4 (KLF4), Nanog homeobox (NANOG), octamer-binding protein 3/4 (POU5F1, OCT 3/4), sex-determining region Y-box 2 (SOX2), paired box 6 (PAX6), cell cycle modulator tumor protein 53 (TP53), and the androgen receptor (AR) were all in the PSA interaction sphere (Figure 7A). The AIM sphere of interaction included markers of macrophage activity and polarization MRC1, CD163, PDGFA, PTGES2, NOS2, CD86, CD80, IL6, TNF, CCR7, FCGR1A, FCGR2A, FCGR3A, ITGAM, biomarkers of T-cell activation and positive response SELL, CD8A, CD69, CLEC7A, TNFRSF4, TNFRSF9, PTPRC, and stemness markers PROM1/CD133 (Figure 7A). Upon sorting by direct interaction, we observed that AIM/CD5L interacts directly with biomarkers of macrophage activity M1 phenotype markers CD163, FCGR2A, FCGR3A, CD68, M2 phenotype markers MRC1/CD206, CD68, inflammatory cascade signals CRP, SNAI2, T-cell activation markers CD8A, CD69, CD86, CD22, TNFRSF9, and androgen signaling AR (Figure 7B, left; see also Supplementary Table S1). In parallel, PSA/KLK3 interacts directly with androgen signaling AR, stemness marker NANOG, metastasis markers FN1, HGF, A2M, and regulator of cell cycle progression TP53 (Figure 7B, right; also see Supplementary Table S1). Understanding that the co-expression and/or co-localization of proteins may be indicative of functional interaction, we further investigated the possibility and nature of AIM-PSA interaction. Using the Schrödinger^®^ PyMOL molecular graphics software version 2.3.2 (https://pymol.org/2/, accessed on 14 February 2021), we created a visualization of the molecular interaction between AIM/CD5L (NCBI Reference Sequence: NP_005885.1) and PSA/KLK3 (NCBI Reference Sequence: NP_001639.1), thus showing that AIM directly targets and binds to PSA with a ligand root-mean-square deviation (RMSD) of 61.13 Å, docking score of 15194, AIM/PSA complex interface area of 2198.90 Å^2^, and an atomic contact energy (ACE) of −282.37 kcal/mol (Figure 7C). The ligand transformation that transforms PSA/KLK3 onto the receptor, AIM/CD5L consists of three rotational parameters, namely −0.27, −0.38, 0.49, and three translational parameters, −70.70, 91.55, 1.57. The interface residues within 5.0 Å from their interacting partner or each other, and the corresponding distances are shown in Supplementary Table S2. In corroboratory experiments, using the co-immunoprecipitation (co-IP) assays, we validated the direct interaction between AIM and PSA (Supplementary Figure S1). These data suggest that AIM binds directly to PSA, and the AIM-PSA interactome is complicit in the immune landscape, macrophage activity, EMT, and CSC regulation.

### 3.8. Ectopic (Re)Expression of AIM Enhances the Anticancer Therapeutic Effect of Pembrolizumab against PCa Cells

Having demonstrated the critical role of the AIM/PSA signaling in prostate cancerization, anticancer immune response, disease progression, and prognosis, we investigated the therapeutic actionability of AIM using the gain-of-function approach (PC3_CD5L_OE) with or without treatment with the anti-PD1 agent, Pembrolizumab (Pembro). Compared with the PC3_WT, 5 μM Pembro moderately suppressed the proliferation of PC3 cells (day 5: 1.26-fold, *p* < 0.05), this was even more so with PC3_CD5L_OE as shown by a 1.77-fold suppressed cell proliferation (*p* < 0.01). Interestingly, when combined with CD5L_OE (PC3_CD5L_OE + Pembro), the anti-proliferative effect of Pembro was significantly enhanced compared to Pembro alone (3.42-fold, *p* < 0.01), or vehicle-treated PC3_WT (4.34-fold, *p* < 0.001) (Figure 7D). Moreover, in comparison with the PC3_WT cells, we observed a 3.85-fold (*p* < 0.01), 1.41-fold (*p* < 0.05), and 11.9-fold (*p* < 0.001) reduction in the number of invaded PC3_CD5L_OE, PC3_WT + Pembro, and PC3_CD5L_OE+Pembro cells, respectively (Figure 7E). Similarly, compared with the vehicle-treated and pembro-treated wild-type cells, tumorsphere formation was profoundly attenuated in the PC3_CD5L_OE and PC3_CD5L_OE + Pembro cells, quantitatively and tumorsphere size-wise (Figure 7F). Of therapeutic relevance, we also found that compared to patients who were not respondent to Pembro from our in-house PCa cohort, the Pembro_responders exhibited significantly higher levels of AIM/CD5L and PDL1 proteins expression, while conversely, PSA/KLK3 protein expression was higher in the Pembro_non-responders (Figure 7G). We also observed that the CD8^+^ T-cell population per visual field was significantly more in the AIM^hi^PSA^lo^PDL1^pos/hi^ Pembro_responders than in the AIM^lo^PSA^hi^PDL1^pos/lo-neg^ Pembro_non-responders (Figure 7H). These data do indicate that the AIM is an actionable molecular factor, and that the ectopic (re)expression of AIM enhances the anticancer therapeutic effect of Pembrolizumab against PCa cells.

## 4. Discussion

Contextualized in the controversial use of PSA as a PCa screening tool, in terms of biomarker hypersensitivity, overdiagnosis, and over-treatment [7,8,9], and the reported role of AIM in host innate and adaptive immunity, inflammatory response regulation, and immune cell viability [10,11,12], the present study explored and provides preclinical evidence of the interaction between AIM and PSA, as well as the putative role of AIM in the modulation of PSA activity, cancerization, disease progression, anticancer therapy response, and prognosis.

Herein, we demonstrated that the expression profiles of AIM and PSA are inversely correlated in patients with PCa, and that concomitant aberrant expression of PSA with significantly suppressed AIM expression characterizes advanced stage prostate cancer, regardless of mutation status (Figure 1 and Figure 2). This is in part consistent with contemporary knowledge that PSA levels are high in PCa cells, and that this is even more so in the advanced stage or high-grade PCa, wherein the PSA level is usually elevated [7,8,19,20]. More so, high levels of AIM have been reported in normal hepatocytes, while in contrast, AIM accumulated on the surface of cancerous liver cells inhibits regulators of complement activation, activates the innate immune system complement cascade, and consequently induce necrosis/necroptosis of the AIM-bound cancerous liver cells [21]. In line with our finding, evidence abounds showing that AIM-deficient mice were highly predisposed to hepatocellular carcinoma, while no AIM-enriched litter-mate developed liver cancer [21].

Cognizant of the usually long course of PCa, and the increasing prevalence of metastatic disease, as well as the high risk of local recurrence or progression to metastatic disease, and eventually death, despite initial definitive local treatment [1,2,3], it is of translational relevance that we found that a high PSA/AIM expression ratio is associated with enhanced metastability, and disease recurrence in patients with PCa, whereas, a high AIM/PSA ratio is associated with strong castration-induced regression (Figure 3 and Figure 4). Concordant with these findings, in a recent study that prospectively evaluated six candidate biomarkers for detection of pelvic lymph node (LN) metastases (pN1) and prediction of BRFS in treated patients, while KLK2 and KLK3 outperformed the other predictive variables, and correctly classified all pN1 cases as molecular node-positive, KLK3 exhibited the highest concordance (96%) with histopathology for detection of LN metastases in the patients with PCa [22,23], and KLK3 protein expression was significantly enhanced in recurrent PCa tissues compared to the ‘normal’ tissues [24]. Moreover, Sugisawa et al. [25] attributed the elimination of HCC cells to the AIM/CD5L produced by liver stellate macrophages/Kupffer cells. They opined that the “blood AIM released from IgM contributes to suppression of obesity and fatty liver as in AKI, whereas macrophage-derived non-circulating AIM mainly prevents HCC development” [25].

Furthermore, corollary to published reports indicating that by binding to human regulator of complement activation (RCA), and blocking the RCA activity, AIM/CD5L facilitates the immune recognition of cancer cells [21], we also demonstrated that not only does the functional association between PSA and AIM modulate metastasis, but that it mirrors the immunogenicity, and differentially modulate Treg, T-cell and macrophage activities in patients with PCa (Figure 5 and Figure 6). Corroborating our findings, it has been demonstrated that AIM/CD5L-deficient mice exhibited reduced T-cell and NKT cell populations in hepatic granulomas unlike their WT peers challenged with heat-killed *C. parvum* [26]. Premised on reports that T-cell-mediated anticancer immune response is regulated by a cascade of co-stimulatory and co-inhibitory signals, with PCa cells exploiting the co-inhibitory signals for evasion of immunosurveillance [27], our present study results indicate that, unlike *PSA/KLK3* which is upregulated, *AIM/CD5L* is concomitantly suppressed with markers of T-cell activation *CD8A*, CD28, *TNFRSF9*, *CD69*, *PTPRC*, *SELL*, and *CCR7* mRNA in primary localized and castration-resistant bone metastatic PCa, but markedly enhanced in normal samples. This probably explains the acquisition and/or maintenance of metastatic, castration-resistant phenotype of the PCa cells, as an effective anticancer immune response requires T-cell activation and co-stimulation by the B7 ligand and CD28 receptor families on antigen-presenting cells (APC) and T cells, respectively [27]. We posit that PSA skews the immune landscape towards immunosuppression by suppressing AIM expression and acting as a co-inhibitory signal which represses T-cell activation, with consequent elicitation of T-cell exclusion, exhaustion, or tolerance in patients with metastatic or recurring PCa. Co-expressed with classifiers of central memory T cells (T_CM_), namely *CD8A*, *CD28*, *PTPRC*, *SELL*, and *CCR7,* it is probable that *AIM* plays a vital role in the characteristic rapid differentiation of the T_CM_ into effector memory (T_EM_) and terminal effector (T_EF_) T cells, all of which are relevant to effective immunotherapy [28,29].

Furthermore, our data indicate that in bone metastatic CRPC, *AIM/CD5L,* and M1 macrophage markers *CD86, FCGR2A, FCGR1A, ITGAM, FCGR3A, CD80*, were co-downregulated, while *PSA/KLK3,* and M2 macrophage markers *MRC1/CD206, CD163* were concurrently upregulated (Figure 6). Tumor-associated macrophages (TAMs) constitute a large portion of the tumor-infiltrating immune cells and play critical but divergent roles in immunosuppression, cancer metastasis, and progression, depending on the phenotype. A recent review of about 300 studies on the prognostic implication of infiltrated M1 or M2 macrophage subtypes, concluded that while the M2 macrophages are associated with poor prognosis, the presence of the M1 macrophages corresponds with a favorable clinical outcome [30]. It is conceivable that by enhancing the recruitment of CD206^+^CD163^+^ M2 macrophages, PSA facilitates an immunosuppressive TME characterized by upregulated Treg and suppressed dendritic cell pooling, extracellular matrix remodeling, and upregulation of “don’t eat me” signals [31]. Conversely, we posit that AIM, by enhancing the infiltration of CD80^+^CD86^+^ M1 macrophages, activates adaptive immunity, enhances antigen presentation, represses the “don’t eat me” signal, and thus, reactivates anticancer immune activity [30,31]. This would be consistent with the concomitant expression of AIM with FCGR2A, FCGR1A, ITGAM, and FCGR3A, all of which play essential roles in enhanced anticancer immune responses and are associated with good clinical outcomes [32,33,34,35].

Moreover, it is clinically enigmatic that a large percentage of advanced PCa cases recur after initial androgen deprivation therapy (ADT), with enhanced risk of progressing to lethal metastatic CRPC; and while immune checkpoint inhibition with anti-CTLA4 or anti-PD1/PDL1 elicits durable response/remission in many cancer types, there is accruing evidence it does not in advanced PCa, metastatic CRPC inclusive [36,37]. We consider our data demonstrating that the ectopic (re)expression of AIM enhances the anticancer therapeutic effect of Pembrolizumab against PCa cells (Figure 7) to be of therapeutic relevance, and consistent with the emerging therapeutic paradigm of combining immune checkpoint inhibition with targeted therapies in the treatment of advanced PCa or CRPC. Interestingly, we demonstrated that the CD8^+^ T-cell population was significantly more in the AIM^hi^PSA^lo^PDL1^pos/hi^ Pembro_responders than in the AIM^lo^PSA^hi^PDL1^pos/lo-neg^ Pembro_non-responders. This is clinically relevant because “although PDL1 positivity enriches for populations with clinical benefit, PDL1 testing alone is insufficient for patient selection in most malignancies” [38] and concordant with the recent report that immunotherapy responders showed higher percentages of T cells positive for ICOS and PD-1 prior to immunotherapy initiation [39].

## 5. Conclusions

Herein, we demonstrate that AIM/CD5L binds to PSA and that a high PSA/AIM ratio, which defines advanced stage PCa (regardless of mutation status), is implicated in enhanced metastability, and is associated with disease recurrence, while a high AIM/PSA ratio is associated with strong castration-induced regression. More so, the ectopic expression of AIM significantly enhances the anticancer effect of the anti-PD1 therapeutic antibody, Pembrolizumab, and elicits an increased CD8^+^ T-cell count in AIM^hi^PSA^lo^PDL1+ PCa cases that are respondent to Pembrolizumab treatment. This study lays the groundwork for future clinical trials on the prognostic accuracy of the AIM/PSA ratio, and further exploration of the clinical feasibility of immune checkpoint blockade and AIM agonist combinatorial therapy as a therapeutic strategy in the clinical management of patients with advanced stage PCa.

## Data Availability

The datasets used and analyzed in the current study are publicly accessible as indicated in the manuscript.

## Supplementary Materials

The following are available online at https://www.mdpi.com/article/10.3390/biomedicines9091225/s1, Supplementary Figure S1. Co-immunoprecipitation (co-IP) data showing the interaction between PSA and AIM in PC3 and DU145 cells. Protein-protein interactions were immunodetected using PSA and AIM monoclonal antibodies. Supplementary Table S1. CD5L/AIM and KLK3/PSA interactome. Supplementary Table S2. AIM-PSA complex interface residues.
