# Supplementary material for: Concomitant High Apoptosis Inhibitor of Macrophage (AIM) and Low Prostate-Specific Antigen (PSA) Indicates Activated T Cell-Mediated Anticancer Immunity, Enhance Sensitivity to Pembrolizumab, and Elicit Good Prognosis in Prostate Cancer
Source: Biomedicines. 2021 Sep 15;9(9):1225. doi: 10.3390/biomedicines9091225 (PMC8469063; doi:10.3390/biomedicines9091225)
Supplement: Supplementary file 1 [file biomedicines-09-01225-s001.zip › biomedicines-1335765-supplementary.pdf]

biomedicines

MDPI

Concomitant High Apoptosis Inhibitor of Macrophage (AIM) and Low Prostate-Specific Antigen (PSA) Indicates Activated T Cell-Mediated Anticancer Immunity, Enhance Sensitivity to Pembrolizumab, and Elicit Good Prognosis in Prostate Cancer

Table S1. CD5L/AIM and KLK3/PSA interactome.

**CD5L interactome**		**KLK3 interactome**	
MRC3 (mannose receptor C-type 1)	Mediates the endocytosis of glycoproteins by macrophages.<br><br>Acts as phagocytic receptor for bacteria, fungi and other pathogens.	FN1<br>(Fibronectin 1)	Involved in cell adhesion, cell motility, opsonization, wound healing, blood coagulation, maintenance of cell shape, host defense, and metastasis.
CRP<br>(C-reactive protein)	Involved in complement activation and amplification via communication with<br>complement initiation pattern recognition molecules.<br><br>Involved in complement regulation via recruitment of complement regulators.<br><br>It is involved in several host defense-related functions via its ability to recognize foreign pathogens and damaged host cells; initiating their elimination by interacting with humoral and cellular effector systems in the blood.	HGF<br>(Hepatocyte Growth Factor)	Secreted by mesenchymal cells and acts as a multifunctional cytokine on cells of mainly epithelial origin.<br><br>Binds to the hepatocyte growth factor receptor to regulate cell growth, cell motility and morphogenesis in numerous cell and tissue types.<br><br>Plays a role in angiogenesis, tumorigenesis, and tissue regeneration.
SNAI2<br>(Snail family transcriptional repressor 2)	Transcriptional repressor that binds to E-box motifs Involved in epithelial-mesenchymal transitions and has anti-apoptotic activity.	A2M (Alpha-2-Macroglobulin}	A protease inhibitor and cytokine transporter Inhibits inflammatory cytokines, thus disrupting inflammatory cascades.

			Inhibits all 4 classes of proteinases by a unique 'bait-and-trap' mechanism.
AR<br>(Androgen receptor)	Functions as a steroid hormone activated transcription factor.<br><br>Stimulates transcription of androgen responsive genes.	AR<br>(Androgen receptor)	Functions as a steroid hormone activated transcription factor.<br><br>Stimulates transcription of androgen responsive genes.
CD68<br>(Macrophage Antigen CD68)	Highly expressed by human monocytes and tissue macrophages.<br><br>Clears cellular debris, promote phagocytosis, and mediate the recruitment and activation of macrophages.	TP53<br>(Tumor Protein P53)	Induces cell cycle arrest, apoptosis, senescence, DNA repair, or changes in metabolism.
CD69<br>(Early T-Cell Activation Antigen CD69)	Involved in lymphocyte proliferation and functions as a signal transmitting receptor in lymphocytes, natural killer (NK) cells, and platelets.	NANOG<br>(Nanog Homeobox)	Involved in embryonic stem (ES) cell proliferation, renewal, and pluripotency.<br><br>Blocks ES cell differentiation and can also auto-repress its own expression in differentiating cells.
CD86<br>(T/B-Lymphocyte Activation Antigen CD86)	Binding of this protein with CD28 antigen is a costimulatory signal for T-cell activation and proliferation.<br><br>Binding of this protein with cytotoxic T-lymphocyte- associated protein 4 negatively regulates T-cell activation and diminishes the immune response.		
CD22<br>(T-Cell Surface Antigen/B-Cell Receptor CD221	Mediates B-cell B-cell interactions.		

	Plays a role in positive regulation through interaction with Src family tyrosine kinases.<br><br>May act as an inhibitory receptor by recruiting cytoplasmic phosphatases via their SH2 domains that block signal transduction through de-phosphorylation of signaling molecules.		
CD163<br>(Macrophage-Associated Antigen CD163)	Exclusively expressed in monocytes and macrophages.<br>An acute phase-regulated receptor involved in the clearance and endocytosis of hemoglobin/haptoglobin complexes by macrophages.<br><br>Protect tissues from free hemoglobin-mediated oxidative damage.<br><br>Function as an innate immune sensor for bacteria and inducer of local inflammation.		
CD8A<br>(T Lymphocyte Differentiation Antigen CD8A)	Found on most cytotoxic T lymphocytes that mediates efficient cell-cell interactions within the immune system.<br><br>Acts as a co-receptor with the T-cell receptor on the T lymphocyte to recognize antigens displayed by an antigen presenting cell in		

	the context of class 1 MHC molecules.		
FCGR2A<br>(Fc Fragment of IgG Receptor Ila)	Found on the surface of many immune response cells, such as macrophages and neutrophils.<br><br>Involved in the process of phagocytosis and clearing of immune complexes.<br><br>Promotes phagocytosis of opsonized antigens.		
FCGR3A<br>(Fc Fragment of IgG Receptor Illa)	Expressed on natural killer (NK) cells as an integral membrane glycoprotein anchored through a transmembrane peptide.<br><br>Involved in the removal of circulating antigen-antibody complexes.<br><br>Mediates antibodydependent cellular cytotoxicity (ADCC) and other antibody dependent responses, such as phagocytosis		
TNFRSF9<br>(Tumor Necrosis Factor Receptor Superfamily Member 9)	Its expression is induced by lymphocyte activation.<br><br>Contributes to the clonal expansion, survival, and development of T-cells.<br><br>Induces proliferation in peripheral monocytes, may enhance T cell apoptosis induced by TCR/CD3- triggered activation, and regulates CD28 co-stimulation		

	to promote Th1 cell responses.<br><br>Facilitates activation of NF-kappaB.		

Table S2. AIM-PSA complex interface residues.

Receptor interface residue(s)			Ligand interface residue(s)			Receptor-ligand interface residue pair(s)	
ARG	36A	2.862	ARG	47A	2.817	36A —107A	3.390
GLU	38A	4.273	ILE	67A	4.365	36A —109A	2.862
GLU	40A	2.817	ARG	68A	4.002	38A —109A	4.273
GLN	44A	3.196	ASN	69A	4.025	40A —47A	2.817
TRP	45A	3.419	LYS	70A	3.782	44A —165A	3.196
GLY	46A	4.305	VAL	92A	3.743	45A —47A	3.419
THR	47A	3.643	SER	93A	2.820	45A —109A	3.659
TYR	79A	4.251	HIS	94A	3.041	46A —109A	4.305
GLU	80A	4.399	SER	95A	2.681	47A —109A	3.643
PRO	81A	3.017	PHE	96A	2.046	79A —107A	4.251
PRO	82A	2.764	PRO	97A	2.650	80A —108A	4.399
LYS	85A	4.210	HIS	98A	3.885	81A —108A	4.755
GLU	118 A	4.776	PRO	99A	2.477	81A —109A	3.017
LYS	173A	3.050	LEU	100A	3.634	82A —109A	4.634
ARG	177A	2.681	TYR	101A	4.920	82A —110A	2.764
GLY	180A	4.365	MET	103A	4.365	85A —109A	4.210
CYS	181A	4.312	LYS	107A	3.390	118A —108A	4.776
GLY	182A	4.124	ASN	108A	4.399	118A —112A	4.988
ARG	183A	2.046	ARG	109A	2.862	173A —94A	3.050
GLY	210A	3.041	PHE	110A	2.764	173A —96A	3.600
ARG	211A	2.820	ARG	112A	4.988	173A —260A	3.782
ALA	213A	4.025	ASP	116A	2.225	177A —67A	4.365
GLU	239A	4.032	SER	118A	2.866	177A —68A	4.002
ASP	240A	2.477	HIS	119 A	3.944	177A —69A	4.050
HIS	261A	3.812	LEU	123A	4.646	177A —93A	4.587
LYS	262A	3.617	PHE	165A	3.196	177A —94A	3.195
VAL	305A	4.567	THR	193A	3.563	177A —95A	2.681
HIS	327A	3.634	LYS	194A	2.613	180A —99A	4.535
ARG	328A	2.225	PHE	195A	3.167	180A —101A	4.920
PHE	329A	3.768	HIS	248A	4.164	180A —103A	4.365
TRP	330A	2.970	LYS	251A	2.970	181A —99A	4.312
GLY	331A	3.356	TRP	252A	2.978	182A —97A	4.374
PHE	332A	4.435	ASP	255A	4.884	182A —99A	4.124
HIS	333A	3.609	THR	256A	4.359	183A —96A	2.046

ASP	334A	2.613	ASN	260A	3.782	183A −97A	2.650
						183A −98A	3.885
						183A −99A	2.881
						183A −123A	4.646
						183A −252A	2.978
						183A −256A	4.359
						210A −93A	4.735
						210A −94A	3.041
						211A −70A	3.782
						211A −92A	3.743
						211A −93A	2.820
						211A −94A	4.483
						213A −69A	4.025
						239A −99A	4.032
						240A −99A	2.477
						240A −100A	4.481
						261A −99A	4.903
						261A −100A	3.812
						262A −98A	4.533
						262A −100A	4.708
						262A −252A	3.617
						305A −251A	4.567
						327A −100A	3.634
						328A −116A	2.225
						328A −118A	2.866
						328A −119A	3.944
						328A −193A	3.563
						328A −194A	4.677
						328A −195A	3.167
						329A −100A	3.768
						329A −195A	4.676
						330A −251A	2.970
						330A −255A	4.884
						331A −194A	3.356
						332A −194A	4.435
						333A −194A	3.609
						333A −248A	4.164
						334A −194A	2.613
